# Early Bedside Risk Stratification in ICU Patients with Suspected Invasive Pulmonary Aspergillosis: A Minimalist Clinical Decision Support Model

**DOI:** 10.3390/jof12090658

**Published:** 2026-09-02

**Authors:** Andrea Baldi, Giulia Capecchi, Francesca Fiani, Antonio Lesci, Dorotea Rubino, Giulia Valeria Stazi, Elena Mattiucci, Valerio Sabatini, Daniele Guerino Biasucci, Carla Fontana, Christian Napoli, Maria Grazia Bocci

**Affiliations:** 1 Department of Computer, Control and Management Engineering, Sapienza University of Rome, 00185 Roma, Italy; baldi.1966232@studenti.uniroma1.it (A.B.); fiani@diag.uniroma1.it (F.F.); cnapoli@diag.uniroma1.it (C.N.); 2 Intensive Care Unit, Clinical and Research Department, National Institute for Infectious Diseases “Lazzaro Spallanzani”—IRCCS, 00149 Rome, Italy; dorotea.rubino@inmi.it (D.R.); giuliavaleria.stazi@inmi.it (G.V.S.); elena.mattiucci@inmi.it (E.M.); valerio.sabatini@inmi.it (V.S.); mariagrazia.bocci@inmi.it (M.G.B.); 3 Istituto di Anestesiologia e Rianimazione, Università Cattolica del Sacro Cuore, 00161 Rome, Italy; antonio.lesci01@icatt.it; 4 Department of Clinical Science and Translational Medicine, University of Tor Vergata, 00133 Rome, Italy; biasucci@med.uniroma2.it; 5 Laboratory of Microbiology and Biobank, National Institute for Infectious Diseases “Lazzaro Spallanzani”—IRCCS, 00149 Rome, Italy; 6 Institute for Systems Analysis and Computer Science, Italian National Research Council, 00185 Rome, Italy; 7 Department of Computational Intelligence, Czestochowa University of Technology, 42-201 Czestochowa, Poland

**Keywords:** invasive pulmonary aspergillosis, predictive model, ICU, Logistic LASSO regression

## Abstract

**Background**: Early diagnosis of Invasive Pulmonary Aspergillosis (IPA) in non-neutropenic ICU patients remains challenging due to the low specificity of radiological findings and the limited reliability of individual biomarkers. This diagnostic uncertainty often delays antifungal treatment and contributes to the high mortality associated with IPA. We aimed to develop a clinically interpretable model for early bedside risk stratification. **Methods**: We conducted a retrospective study including 298 ICU patients. A Logistic LASSO regression approach was used to identify the most informative predictors of IPA and to develop a parsimonious predictive model. Model performance was assessed through internal exploration and robustness testing under noise perturbation. **Results**: Three routinely available clinical variables were retained in the final model: pulmonary galactomannan, non-specific pulmonary infiltrates, and patient age, reflecting fungal burden, lung involvement, and host vulnerability, respectively, and identifying patients with a higher probability of culture-positive *Aspergillus* detection within a clinical context compatible with IPA. The final model showed promising discriminative performance, with an AUC of 0.836 ± 0.075, sensitivity of 0.72, and specificity of 0.74. Despite relying on only three routinely available clinical variables, the model maintained stable performance during internal exploration and robustness analyses. **Conclusions**: Within this cohort, the combination of positive pulmonary galactomannan, radiological infiltrates, and advanced age identifies a subgroup of ICU patients with a higher probability of microbiological positivity and clinical suspicion of IPA, supporting early risk assessment rather than definitive diagnosis. This simple and transparent predictive model may support early clinical evaluation and risk stratification of ICU patients and assist clinicians in identifying patients requiring further diagnostic assessment.

## 1. Introduction

Invasive Pulmonary Aspergillosis (IPA) has traditionally been associated with profoundly immunocompromised patients, particularly those with neutropenia [1]. However, in recent years, its epidemiology has shifted significantly, with an increasing number of cases observed in non-neutropenic patients admitted to Intensive Care Units (ICUs), including those with severe viral pneumonia, chronic obstructive pulmonary disease, or advanced liver disease [1,2,3,4]. In this setting, IPA represents a frequently under-recognized complication associated with mortality rates reaching nearly 50% [5].

The central clinical challenge lies in the difficulty of establishing a timely and reliable diagnosis. In ICU patients, the classical diagnostic framework for IPA becomes problematic. Histopathological confirmation, although regarded as the diagnostic gold standard for IPA, is rarely feasible in critically ill patients because lung biopsy is often unsafe or impractical. Moreover, even when tissue sampling is performed, limited or non-representative sampling may reduce diagnostic yield and potentially lead to false-negative results [6,7]. As a result, clinicians are frequently forced to rely on indirect evidence. This fact exposes important limitations. Biomarkers such as galactomannan (Gal), as well as conventional radiological signs, demonstrate reduced sensitivity and specificity in non-neutropenic patients compared to hematological populations [8]. Typical features such as the “halo sign” are uncommon, while radiological findings are often nonspecific and overlap with those of underlying lung diseases or concurrent infections. Consequently, IPA in ICU patients is characterized by a diagnostic uncertainty, where neither clinical, microbiological, nor imaging findings alone are sufficient to support confident decision-making.

This ambiguity has direct clinical consequences [9]. Delayed or missed diagnosis can lead to late initiation of antifungal therapy, while overdiagnosis may expose patients to unnecessary treatments and toxicity. Therefore, there is a clear need for strategies that can support clinicians in identifying patients at higher risk of IPA using information that is readily available in routine practice.

In this context, the present study focuses on the development of a clinically oriented predictive approach based on a limited number of variables. 298 ICU patients were screened for *Aspergillus* infection with only 25 culture-based confirmed cases. This reflects the reality of specialized clinical research, where data are highly informative but limited in size.

Rather than relying on high-dimensional models requiring extensive datasets, we aimed to identify a stable and interpretable clinical decision-support model.

This methodological approach was guided by careful clinical and statistical evaluation of candidate variables, with particular attention to redundancy and confounding between inflammatory markers. Emphasis was placed on selecting features that are both clinically meaningful and consistently available at the bedside.

## 2. Materials and Methods

### 2.1. Setting

The National Institute for Infectious Diseases ‘Lazzaro Spallanzani’ is a healthcare and research institute, specialized in the management and care of complex infectious disease patients.

### 2.2. Study Design, Patient Selection and Diagnostic Work-Up

This is an observational, monocentric, retrospective study conducted at ICU of the National Institute for Infectious Diseases “Lazzaro Spallanzani” (IRCCS), Rome, Italy. Data were collected from January 2021 to December 2025. The study cohort retrospectively included 298 adult patients (age >18 years) admitted to the ICU who underwent an *Aspergillus* diagnostic work-up because of clinical suspicion of IPA. Clinical suspicion was raised in patients presenting with unexplained clinical deterioration despite appropriate antibacterial therapy and/or radiological findings suggestive of pulmonary fungal infection. Therefore, the study population did not include all ICU admissions but rather those in whom IPA was considered clinically plausible and a dedicated microbiological investigation was initiated. Patients meeting these criteria during the study period constituted the analytical cohort. Patients and public were not involved in the design, conduct, reporting, interpretation or dissemination of the study.

According to the institutional diagnostic protocol, once IPA was clinically suspected, bronchoalveolar lavage (BAL) was performed. Fungal culture, *Aspergillus* PCR, and galactomannan testing were requested simultaneously from the same BAL sample as part of the routine diagnostic work-up. Radiological findings could either contribute to the initial clinical suspicion or be obtained during the diagnostic evaluation. Consequently, all candidate predictors included in the model were available during the initial diagnostic assessment, whereas fungal culture positivity became available subsequently and served as the microbiological outcome for model development. Patients were evaluated for suspected IPA according to institutional clinical practice; the study endpoint was fungal culture positivity within this predefined diagnostic pathway and was not intended to replace established consensus definitions of proven or probable IPA.

### 2.3. Ethical Approval

Ethical review and approval were waived for this study by the Ethics Committee (Comitato Etico Territoriale Lazio Area 4; protocol code: 13-2025; approval date: 28 May 2025). Due to its retrospective nature, informed consent was waived. The study adhered to the principles of the Declaration of Helsinki and to the Good Clinical Practice Guidelines.

### 2.4. Dataset Structure and Preprocessing Strategy

Clinical data are heterogeneous, often characterized by a mixture of laboratory measurements and radiological assessments. We opted for a binarization of all features. This decision is rooted in the “Rule-Based Medicine” paradigm, which uses conditional logic derived from clinical guidelines and expert knowledge to process patient data and automate medical decisions [10]. Binarization enhances model stability in small datasets by mitigating the impact of outliers and reducing the noise typical of clinical records. To avoid selection bias, we verified that diagnostic tests, such as PCR and Gal, were performed on all patients in the cohort, regardless of their initial risk profile. Originally, the raw dataset contained 72 variables with a wide number of different analyses. Accurate manual preprocessing was required to keep just informative values, to create a clear binarized dataset avoiding any type of data leakage. Detailed definitions and binarization criteria for all candidate predictors are reported in Supplementary Table S1.

The output expected is a binarized and noise-reduced dataset suitable for our statistical evaluations and LASSO pipeline. To achieve the complete binarization of the dataset variables, the variable Age was dichotomized at ≥65 years as a pragmatic and clinically interpretable marker of older age. This cutoff should not be interpreted as a discrete biological transition. Rather, it was used as a proxy for age-related host vulnerability. This choice is supported by observational evidence showing that patients with invasive aspergillosis tend to be older than those without the disease, and that age ≥65 years is associated with worse outcomes among patients with IPA lacking classical immunocompromised host factors [11,12]. Radiological values are binarized based on the presence of a specific sign in patient radiology, whereas galactomannan was set as positive if the value of galactomannan in the sample exceeded 0.5.

### 2.5. Feature Selection and Data Cleaning

The “Ground Truth” for the classification task was established using the microbiological culture-based results, represented by the variable culture (Col). For the purpose of this retrospective analysis, culture positivity was used to define the microbiological reference outcome. This outcome should be interpreted as a microbiological surrogate and not as definitive evidence of tissue-invasive disease. Although culture provides direct evidence of *Aspergillus* recovery, it does not by itself establish tissue invasion and should therefore be interpreted within the broader clinical, radiological, and mycological context of IPA diagnosis. Accordingly, the selected outcome should be interpreted as a microbiological surrogate rather than definitive evidence of invasive disease, and its use reflects the constraints of retrospective ICU-based diagnostic data. The initial preprocessing phase resulted in a set of 24 original clinical and radiological features. The complete list of binarized predictors, prior to engineering, is as follows:

- Host Factors: sex, age, use of corticosteroids, treatment with cytotoxic agents, history of organ transplant, HIV, hematological/oncological malignancies, immunosuppression, arterial hypertension, diabetes mellitus, COPD, COVID-19 infection, meningitis and encephalitis, refractory respiratory failure despite mechanical ventilation.
- Radiological Signs: air crescent sign, cavity, circumscribed lesion with or without halo sign, alveolar or reticular opacities, non-specific pulmonary infiltrates, pleural effusion, wedge-shaped opacities, tree in bud pattern.
- Laboratory Markers: PCR, Gal.

### 2.6. Feature Engineering

A dedicated Feature Engineering (FE) phase was included. Beyond individual parameters, we hypothesized that the risk of IPA is often defined by the synergistic interaction between biological, radiological, and host-related factors. A single marker might be insufficient for a stable diagnosis; however, its predictive power may increase significantly when combined with a second, correlated signal.

FE_Gal_x_PCR: This term represents the synergy between the specific fungal Gal antigen and PCR assay.

FE_Inf_x_Gal: This interaction combines morphological radiological damage of non-specific Infiltrates (Inf) with biological evidence of the presence of Gal antigen. It represents the integration of microbiological antigen detection with radiological lung abnormalities, reflecting a combined biological–morphological pattern that is frequently associated with clinical suspicion of IPA.

FE_Age_x_Immuno: This term focuses on the host vulnerability, exploring how the combination of age and immunosuppressive status (Immuno) amplifies the risk.

These engineered variables encode clinically meaningful interactions between candidate predictors, enabling the LASSO algorithm to evaluate combined patterns of association that may not be captured by univariate analyses alone. The transition from a raw dataset to a predictive model requires a preliminary analysis.

### 2.7. Statistical Analysis and Model Development

In datasets characterized by a limited number of positive events, 25 culture-positive *Aspergillus* cases out of 298 patients, the ratio of features to events often leads to overfitting. High number of variables increases the “noise”, allowing the model to memorize random patterns rather than learning true biological relationships. We conducted an exhaustive univariate and bivariate analysis to establish the baseline predictive power of each feature and map the correlations. Each of the 27 engineered features was evaluated against the Col target using cross-tabulation. Bonferroni-adjusted alpha threshold (*p* < 0.00179) was utilized on the whole dataset only as a descriptive reference. We categorized the features into predictive tiers based on their Odds Ratios (ORs) and statistical significance using thresholds not corrected by Bonferroni adjustment.

### 2.8. Iterative Feature Selection and Collinearity-Guided Model Refinement

Given the limited number of outcome events and the high dimensionality of the candidate feature set, a stepwise, collinearity-guided feature refinement strategy was implemented before final model selection. Candidate predictors were initially ranked according to their univariate association with IPA. Variables with stronger univariate signal, clinical plausibility, and potential biological relevance were retained for multivariable modelling.

Because several predictors represented overlapping clinical or biological constructs, multicollinearity was systematically assessed before and during model development. Pairwise Pearson correlation coefficients were calculated among candidate variables, and correlation clusters were identified using a predefined threshold of r > 0.70. In addition, the Variance Inflation Factor (VIF) was computed for each candidate predictor to quantify redundancy within the multivariable feature space. Variables with elevated VIF values, unstable coefficients, or substantial overlap with their base components were considered at risk of introducing coefficient instability and reducing model interpretability.

To address the low prevalence of the outcome and reduce overfitting, logistic regression with L1 regularization was selected as the primary modelling framework. The L1 penalty was used to promote sparsity and support feature selection while preserving interpretability. However, because LASSO may behave unstably in the presence of highly correlated predictors, automated penalization was not used as the sole selection criterion. Instead, LASSO was combined with an iterative pruning strategy based on three complementary criteria: VIF values, Pearson correlation structure, and coefficient stability across validation folds.

The initial model included the top-ranked predictors emerging from exploratory analysis, including baseline clinical variables, radiological findings, microbiological biomarkers, and engineered interaction terms. After each model iteration, candidate variables were evaluated for their contribution to model performance, coefficient direction and magnitude, variability across folds, and residual collinearity. Variables were removed when they showed one or more of the following characteristics: high collinearity with other predictors, unstable or counterintuitive coefficient behavior across cross-validation folds, negligible penalized coefficients, or limited incremental contribution to discrimination. This process continued until a parsimonious and clinically interpretable model with stable coefficients and minimal residual collinearity was obtained.

Model development and internal validation were performed using nested cross-validation to reduce optimism and prevent data leakage. The inner loop consisted of a 3-fold stratified cross-validation used for hyperparameter tuning. The inverse regularization strength parameter, C, was optimized through grid search over a logarithmic range from 0.0001 to 5.0. The outer loop consisted of a 5-fold stratified cross-validation and was used exclusively for performance estimation. All preprocessing procedures, including feature scaling with StandardScaler, were fitted only on the training portion of each fold and then applied to the corresponding validation fold.

To account for the marked class imbalance, with invasive aspergillosis representing a minority outcome, a balanced class-weighting strategy was applied during model fitting. This approach weighted observations inversely proportional to class frequency, thereby reducing the tendency of the classifier to favor the majority class.

Model performance was summarized across the outer validation folds using the area under the receiver operating characteristic curve (ROC-AUC), sensitivity, and specificity, reported as the mean ± standard deviation. Calibration of predicted probabilities (e.g., calibration plots, calibration slope, and Brier score) was not formally assessed due to the limited number of outcomes, which would have led to unstable calibration estimates. Therefore, we position this model as an exploratory internal risk-stratification tool rather than a fully validated clinically deployable probability model. Two different intercept scales were used: standardized scale for LASSO fitting and the original binarized scale for the final equation, with both the intercept and the coefficients derived from the original 0/1 scale. Because balanced class weighting was used during fitting, the model output should be interpreted as a relative risk score rather than an absolute probability of disease occurrence. Coefficient stability was assessed by examining the mean and standard deviation of penalized regression coefficients across the outer folds. Final model selection was based on a combination of discrimination, sensitivity preservation, coefficient stability, low residual collinearity, and clinical interpretability, rather than on ROC-AUC alone.

The full pipeline has been developed partially within the cross-validation folds, and partially on the full cohort. In particular, LASSO fitting, hyperparameter tuning and feature scaling were performed strictly on the folds, whereas preprocessing steps (univariate screening, correlation clustering and pruning) were performed on the full dataset of 298. This is justified by the scarcity of events, which would have rendered per-fold screening statistically unstable. While this choice introduced some degree of performance inflation, all methods presented were evaluated under the same circumstances and are therefore comparable, with their performances evaluated over multiple fold partitions to prove repeatability.

Additional methodological details, including variable binarization criteria, model intercepts and coefficients, the final prediction equation, risk-score thresholds, performance across alternative decision thresholds, and supplementary model stability analyses, are provided in the Supplementary Materials (Supplementary Tables S1–S8 and Figure S1).

### 2.9. Reporting Guidelines

This study was reported in accordance with the Transparent Reporting of a multivariable prediction model for Individual Prognosis Or Diagnosis plus Artificial Intelligence (TRIPOD-AI) guidelines. The completed TRIPOD checklist is provided as Supplementary Table S9.

### 2.10. Use of AI-Assisted Tools

The initial layout and graphical elements of the graphical abstract were generated using FigureLabs (FigureLabs.ai; https://www.figurelabs.ai/; accessed on 23 June 2026), and subsequently manually revised, edited, and assembled by the authors. The authors independently verified the scientific accuracy of all content and took full responsibility for the final graphical abstract.

## 3. Results

### 3.1. Demographic and Clinical Characteristics of the Study Population

The retrospective cohort included 298 critically ill patients, of whom 199 (67%) were male and 99 (33%) were female, with a median age of 62.59 years (IQR 51.50–73.75). At ICU admission, the median white blood cell count was 9.54 × 10^9^/L (IQR 5.34–11.63), with a median neutrophil count of 10.74 × 10^9^/L (IQR 6.07–13.07) and a median lymphocyte count of 0.54 × 10^9^/L (IQR 0.27–0.67). No patient fulfilled the EORTC/MSGERC host-factor criterion for prolonged neutropenia, defined as an absolute neutrophil count <500 neutrophils/mm^3^ for >10 days temporally related to the onset of invasive fungal disease [13]. Regarding underlying conditions, hypertension was present in 112 patients (38%), diabetes mellitus in 55 (18%), and COPD in 35 (12%), while 156 patients (52%) had COVID-19. Immunosuppression was documented in 88 patients (29%). Among immunosuppressed patients, HIV infection was the most frequently represented condition (42%), followed by hematological malignancies (32%), immunosuppressive therapy (22%), and solid-organ transplantation (19%). The relatively high representation of patients living with HIV reflects the specific case mix of our institution as a tertiary referral center specialized in infectious diseases. Baseline demographic, hematological, and clinical characteristics of the study population are summarized in Table 1.

**Table 1:** Baseline characteristics of the study population.

Sex	
Male, *n* (%)	199 (67%)
Female, *n* (%)	99 (33%)
Age, median (IQR)	62.59 (51.50–73.75)
Hematological parameters at admission, median (IQR)	
Neutrophils, ×10^9^/L	10.74 (6.07–13.07)
WBC, ×10^9^/L	9.54 (5.34–11.63)
Lymphocytes, ×10^9^/L	0.54 (0.27–0.67)
Comorbidities, *n* (%)	
COPD	35 (12%)
Diabetes Mellitus	55 (18%)
Hypertension	112 (38%)
COVID-19	156 (52%)
Immunodepression	88 (29%)
Conditions associated with immunodepression, *n*, (%) *	
Organ transplant recipients	17 (19%)
Hematological malignancies	28 (32%)
HIV	37 (42%)
Corticosteroids use	4 (5%)
Immunosuppressive agents use	19 (22%)

* Percentages for conditions associated with immunodepression were calculated among immunodepressed patients (*n* = 88). Categories were not mutually exclusive, as individual patients could have more than one cause of immunodepression.

### 3.2. Univariate Predictor Screening and Collinearity-Guided Feature Selection

As detailed in Table 2, percentages represent the full analytical cohort (N = 298). The strongest univariate predictors (Tier 1) included the engineered interaction terms (FE_Inf_x_Gal and FE_Gal_x_PCR), alongside the isolated baseline biomarkers (Gal and PCR) and morphological signs (Non-specific infiltrates). While traditional radiological signs like the air_crescent_sign showed a high OR (5.4), its wide 95% confidence interval (1.7–17.0) highlights small-cell instability secondary to their extremely low prevalence (5.7%) and low feature prevalence (5.7%, 17/298) and a low total positive outcome events (*n* = 25), limiting their utility due to the limited population sample size. Age emerged as a solid Tier 2 predictor, confirming the clinical hypothesis of host vulnerability. The dataset has a significant class imbalance, with an overall disease prevalence of 8.4% (25/298). However, stratifying the cohort by key covariates revealed stark risk gradients. For instance, the positivity rate jumped from 4.1% in Gal patients to 27.3% in Gal positive patients. Similarly, patients aged >65 years showed a prevalence of 12.2% compared to 4.2% in the younger cohort. Interestingly, the immunosuppression variable, defined according to Bassetti et al., 2021 [13], (i.e., neutropenia with neutrophils < 500/mm^3^ > 10 days, ≥3 weeks use of corticosteroids ≥0.3 mg/kg, HSCT—Hematopoietic Stem Cell Transplantation, hematologic malignancies, solid organ transplant recipient, severe combined immunodeficiency) alone did not effectively stratify the risk (7.0% in positive vs. 9.0% in negative patients), suggesting that, in non-neutropenic ICU patients, traditional markers of systemic immunosuppression may have limited predictive value for invasive aspergillosis when considered alone and should therefore be interpreted in combination with other clinical factors. High correlation among predictors is a primary cause of instability in linear models. We mapped the feature space identifying multicollinearity clusters (Pearson *r* > 0.7). The interactions correlated with their base components (e.g., FE_Inf_x_Gal vs. FE_Gal_x_PCR, *r* = 0.84).

To obtain this, we computed the Variance Inflation Factor (VIF). The FE_Gal_x_PCR interaction triggered a VIF of 6.83 (>5), while basic variables like Gal and PCR maintained acceptable levels (≈3.0). This confirmed that standard logistic regression or unpenalized methods would fail to converge optimally due to standard error inflation. We also conducted a dedicated joint analysis on PCR and Gal to determine their synergy and overlapping diagnostic utility.

As illustrated in Table 3, the dual-positive state acts as a clinical confirmation, elevating the risk to 34.2% with a Likelihood Ratio (LR+) of 5.68. Conversely, the dual-negative state nearly excludes the infection (1.8% prevalence).

The Cohen’s Kappa score of 0.251 indicates a “fair” but low agreement between the two markers. Biologically, this finding suggests that PCR and galactomannan do not provide fully redundant information, but may reflect different biological dimensions of *Aspergillus*-related disease.

**Table 2:** Main predictive features sorted by Univariate OR (Tier 1 and Tier 2).

Feature	OR	95% CI	*p*-Value	Pearson *r*	* Prev., n/N (%) ^a^	Prev. in Feature Present, n/N (%) ^b^	Prev. in Feature Absent, n/N (%) ^c^	Tier
FE_Inf_x_Gal	14.8	[5.8–37.7]	<0.001	0.40	28/298 (9.4)	11/18 (39.3)	14/270 (5.2)	1, strong
FE_Gal_x_PCR	10.7	[4.4–26.1]	<0.001	0.36	38/298 (12.8)	13/38 (34.2)	12/260 (4.6)	1, strong
Gal	8.7	[3.7–20.8]	<0.001	0.32	55/298 (18.5)	15/55 (27.3)	10/243 (4.1)	1, strong
Inf	7.9	[3.2–19.8]	<0.001	0.29	85/298 (28.5)	18/85 (21.2)	7/213 (3.3)	1, strong
PCR	7.1	[2.6–19.6]	<0.001	0.25	118/298 (39.6)	20/118 (16.9)	5/180 (2.8)	1, strong
Air crescent sign	5.4	[1.7–17.0]	0.0056	0.19	17/298 (5.7)	5/17 (29.4)	20/281 (7.1)	1, strong
Age (≥65 years)	3.1	[1.2–8.1]	0.0235	0.14	156/298 (52.3)	19/156 (12.2)	6/142 (4.2)	2, moderate
Halo sign	2.8	[1.2–6.5]	0.0310	0.14	63/298 (21.1)	10/63 (15.9)	15/235 (6.4)	2, moderate

* Prev. = prevalence. ^a,b,c^ All percentages refer to the total analytical cohort (N = 298), with no missing values present. Variables with *p* > 0.05 or OR < 2.0 were classified as Tier 4 (i.e., noise) and were omitted from the table.

**Table 3:** Joint clinical prevalence for PCR and Gal status.

Status *	*N*	Col+ (%)	Clinical Indication
PCR+, Gal+	38	13 (34.2)	Strong Suspicion
PCR+, GAL−	80	7 (8.8)	PCR isolated
PCR−, GAL+	17	2 (11.8)	Gal isolated
PCR−, GAL−	163	3 (1.8)	Low Risk

* The symbol ‘+’ indicates that the variable is present (coded as 1), whereas ‘−’ indicates that the variable is absent (coded as 0). Both *Aspergillus* PCR and galactomannan were performed simultaneously on the same BAL specimen as part of a structured diagnostic work-up.

At the end of the pipeline, we performed a multivariate regression collinearity test. The coefficients for PCR alone (+1.966) and Gal alone (+2.168) remained positive and stable when combined (+1.522 and +1.753, respectively). The absence of sign inversion confirmed that the collinearity is tolerable. The interaction term (PCR × Gal) was not statistically significant (*p* = 0.803), providing no evidence of a synergistic interaction in this cohort. However, given the limited number of positive events, a potential interaction cannot be excluded. While non-linear architectures such as Random Forests and Gradient Boosting were benchmarked, they demonstrated variance degradation and overfitting during validation. Consequently, Logistic Regression with L1 Regularization (LASSO) was selected as the optimal framework, as it balances predictive performance with clinical interpretability through feature selection.

The algorithmic implementation used the liblinear solver, optimized for coordinate descent in sparse datasets. To address the class imbalance (8.4% prevalence), a balanced class-weighting strategy was applied to the loss function, ensuring the model remains sensitive to the minority class.

Hyperparameter tuning focused on the inverse regularization strength (*C*), evaluated across a logarithmic scale ranging from 0.0001 to 5.0. To reduce optimism, a Nested Cross-Validation (NCV) framework was adopted. It separates hyperparameter optimization from performance estimation through two distinct loops. The inner loop (3-fold Stratified CV) is dedicated to selecting the optimal *C* value via GridSearchCV, while the outer loop (5-fold Stratified CV) provides an internal estimate of model performance. To maintain the integrity of the process, all preprocessing steps, including StandardScaler, were fitted exclusively on the training folds within the loops.

### 3.3. Iterative Model Refinement and Final Feature Selection

While LASSO is theoretically designed to perform autonomous feature selection, its behavior becomes unstable in the presence of severe multicollinearity, a phenomenon known as the grouping effect limitation [14]. To overcome this structural constraint, we executed a guided iterative pruning process. Rather than relying on automated shrinkage, we monitored the Variance Inflation Factor (VIF), the Pearson correlation matrix, and the stability of the LASSO coefficients across the outer NCV folds. This human-in-the-loop refinement was used to improve model parsimony and clinical interpretability and to reduce the influence of redundant or unstable predictors, while the model’s validity remains to be established through external validation.

### 3.4. The Final Model

Following the first two phases of model refinement: the 8-variable baseline model and the 5-variable intermediate model (Supplemental Material pp. 5–6), the final selection phase established the best model achieved, consisting of the only variables: Inf, Gal, and Age (Table 4).

The ROC-AUC increased to its maximum of 0.836 ± 0.075, confirming that removing the redundant variables actually improved the model’s ability to generalize by eliminating high-variance noise. Sensitivity remained stable at 0.720, and specificity improved to 0.740. Notably, the stability of sensitivity indicates that removing redundant variables did not compromise the model’s ability to detect positive cases. Furthermore, for the final 3-variable model, application to the full cohort (N = 298, prevalence = 8.4%) yielded a Positive Likelihood Ratio (LR+) of 2.77, a Negative Likelihood Ration (LR−) of 0.38, a Positive Predictive Value (PPV) of 20.2% and a Negative Predictive Value (NPV) of 96.8%. The high NPV suggests that the model may be useful for identifying a subgroup of patients at lower risk of culture positivity within the studied cohort, potentially supporting preliminary bedside risk stratification and reducing unnecessary screening in selected patients. However, these findings should be interpreted cautiously given the retrospective single-center design, the limited number of culture-positive outcomes, and the absence of external validation.

The choice of this specific triad was driven by rigorous cross-check evaluations (i.e., ablation studies). To verify the indispensability of each pillar, we tested alternative configurations by substituting the core variables with the next strongest candidate, PCR. Replacing Gal with PCR caused the AUC to collapse to 0.778 ± 0.082. Similarly, replacing Inf with PCR resulted in an AUC drop to 0.809 ± 0.037. These experiments supported that PCR, while statistically significant, provided a less stable and less informative signal after adjustment for the selected variables compared to the primary pillars. The triad was selected as the optimal intersection of a biological biomarker (Gal), a morphological radiological marker (Inf), and a host factor (Age). The LASSO coefficients stabilized into a balanced diagnostic triad: Inf (+0.615 ± 0.160), Gal (+0.608 ± 0.110), and Age (+0.350 ± 0.033), achieving minimal residual collinearity (Max VIF 1.07).

### 3.5. Ablation Studies: Feature Substitution and Markers Validation

We performed a series of ablation studies and feature substitution tests. The primary objective was to evaluate whether the inclusion of PCR, the strongest excluded candidate, could enhance or replace the information provided by the variables Gal, Inf, or Age. As summarized in Table 5, the above-mentioned configuration (AUC = 0.836) achieved the highest discrimination with respect to the others.

Substituting Gal with PCR resulted in a significant drop in ROC-AUC to 0.778 ± 0.082, despite maintaining sensitivity. Similarly, replacing Inf with PCR reduced the AUC to 0.809. These reductions suggest that, although PCR is a statistically significant molecular test for IPA diagnosis, it provided limited additional discriminatory value once the selected biological and radiological variables were included in the model.

*Aspergillus* PCR is an important molecular diagnostic tool for IPA, particularly when performed on bronchoalveolar lavage fluid, but it should not be interpreted as a stand-alone diagnostic marker. Its value is greatest when integrated with clinical, radiological, and other mycological findings, especially Gal. However, our pipeline demonstrates how the PCR variable, in this study, provided limited additional discriminatory value once galactomannan, pulmonary infiltrates, and age were included in the model, likely reflecting partial overlap with other biological and radiological signals rather than independent predictive contribution. Therefore, although PCR was statistically associated with IPA in univariate analysis, its contribution became unstable in multivariable modeling and did not add clinically useful information beyond the final triad of Gal, Inf, and age. Furthermore, removing Age to test a 2-variable biomarker-imaging model (Gal and Inf) yielded a high sensitivity (0.840) but at the cost of overall discrimination (AUC = 0.796) and reduced specificity (0.667). The evaluation of Gal + PCR combo confirmed that without the morphological context of infiltrates or the host factor of age, the model falls into a noisier predictive state (AUC = 0.787).

### 3.6. Clinical Risk Stratification and Patient Clustering

We performed a hierarchical clustering analysis to stratify the patient population into distinct risk tiers: low, medium, and high risk. The clustering analysis was performed on the full dataset as a post hoc descriptive analysis. The transition from the 8-variable baseline to the 3-variable revealed a stability in patient categorization. In the 8-variable phase, the high-risk cluster (*N* = 67) identified 16 true positive cases (23.9% real prevalence). However, the profile was cluttered by secondary markers like PCR (84%) and halo sign, which added complexity without increasing the detection rate.

As we moved to the 5-variable phase and finally to the 3-variable phase (the final model), the clusters achieved mathematical maturity. The 3-variable model maintained the exact same identification power as the 5-variable version, isolating a high-risk cluster of 77 patients containing 17 Col positives (22.1% prevalence). This stability confirms that the redundant variables removed during pruning (i.e., PCR and air_crescent_sign) were not contributing to the fundamental separation of risk. The final clustering provides a roadmap for clinical intervention, as summarized in Table 6.

The High-Risk Cluster is characterized by the convergence of all three pillars: advanced age (88%), extensive non-specific pulmonary infiltrates (81%), and positive Gal (56%).

In this group, the expected probability of high risk of IPA is 0.74 ± 0.11, may support further diagnostic evaluation and consideration of therapy in the appropriate clinical context. Due to the application of class weighting during model fitting, this value reflects an uncalibrated relative risk score optimized for sensitivity rather than an absolute empirical incidence rate, aligning with the observed 22.1% microbiological culture positivity rate. Conversely, the Low-Risk Cluster showed a 0% real prevalence of IPA.

### 3.7. Experiments and Comparative Analysis

Beyond the mentioned pipeline, we conducted a comprehensive benchmarking phase. We mapped the performance of all experimental architecture across the three progressive feature configurations: the 8-variable baseline, the 5-variable intermediate subset, and the 3-variable.

This approach allows us to observe how different algorithmic structures react to dimensionality reduction and noise. The comparative suite included baseline Random Forest (RF), eXtreme Gradient Boosting (XGBoost), Principal Component Analysis coupled with Support Vector Machines (PCA + SVM), and a Hybrid LASSO-Guided RF Ensemble. In particular:

- Random Forest (RF): A bagging ensemble of decision trees, robust against non-scaled data and capable of capturing complex non-linear interactions through Gini impurity partitioning.
- XGBoost: A gradient boosting architecture that iteratively corrects the residual errors of weak learners.
- PCA + SVM: A dimensionality reduction technique followed by a maximum-margin classifier (Radial Basis Function). It projects the dataset into orthogonal components before drawing a non-linear decision boundary. This method is the only one which is not tested on the same variable set. SVM works, respectively, on 8, 5 and 3 PCA orthogonal component’s directions (Supplemental Material pp. 2–3; Supplementary Figure S1).
- Hybrid Ensemble: A Random Forest constrained to use only the features selected by the LASSO iterations, designed to test if non-linear splits on a “clean” subset could outperform linear regression.

### 3.8. Performance Comparison: The Sensitivity Bias

The core comparison between the linear LASSO and the tree-based models reveals a fundamental behavior of machine learning algorithms on imbalanced datasets (8.3% prevalence). When fed with the 8-variable configuration, the Baseline RF matched the LASSO in overall discrimination (AUC 0.836 for RF vs. 0.822 for LASSO). However, the internal distribution of this performance was drastically different.

As reported in Table 7, the RF favored the majority class, achieving a specificity of 0.806 but compromising the minority class with a sensitivity of only 0.560 ± 0.329. In contrast, as the LASSO was pruned to its 3-variable, it stabilized its sensitivity at 0.720 ± 0.110 while maintaining an AUC of 0.836.

This phenomenon is a well-documented flaw of standard Random Forest architectures. As demonstrated by O’Brien and Ishwaran [15], tree-based bagging algorithms naturally skew towards the majority class to minimize global error rates, creating an “overfitting pattern” to the negative class.

The LASSO’s ability to maintain a sensitivity of 0.720 makes it superior for emergency bedside triaging. Throughout all experiments sensitivity is the metric taken in consideration alongside AUC. Indeed, when looking for clinical predictions on potentially deadly illnesses, it is always preferable to reduce the number of unpredicted patients with pathologies rather than having high specificity. Therefore, we privilege the LASSO model which shows higher values and robustness on this metric.

### 3.9. Overfitting Detection via Synthetic Data Decay

**Figure 1:**
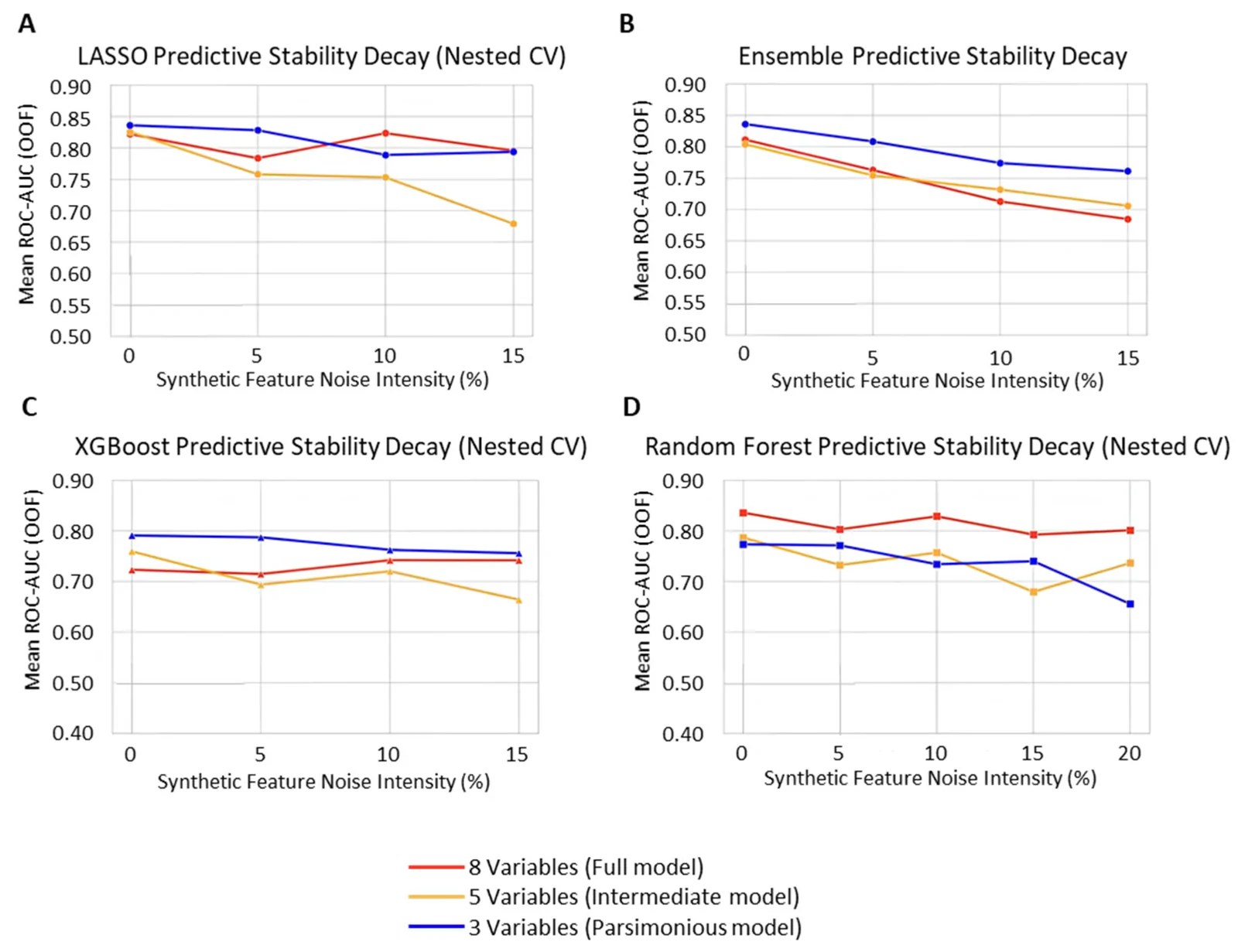
Model performance under synthetic feature perturbation (random bit-flipping of clinical features with probability = 0%, 5%, 10%, 15% for each feature). Changes in mean out-of-fold (OOF) ROC-AUC are shown across increasing levels of synthetic feature noise for (**A**) LASSO, (**B**) Ensemble, (**C**) XGBoost, and (**D**) Random Forest models. Performance is compared across three feature configurations: the Full model (8 variables), Intermediate model (5 variables), and Parsimonious model (3 variables). The analysis provides an additional assessment of model stability under progressive perturbation of the input features.

As an additional assessment of model robustness, we performed a synthetic data perturbation analysis by progressively introducing random noise into the predictor variables. We injected incremental noise (from 0% up to 15%) into the test folds input data during the Nested CV, effectively corrupting the input variables to mimic extreme real-world clinical variances (e.g., laboratory measurement errors or subjective radiological evaluations) (Figure 1).

The decay logs revealed architectural vulnerabilities in some non-linear models. For instance, the Random Forest at 5 variables collapses, dropping from an AUC of 0.787 at baseline to 0.679 at 15% noise even though it doesn’t show significant decay on 8 variables configuration. XGBoost has unstable behavior, starting from a low 0.723 (8 variables) and failing to establish a stable plateau across configurations.

Conversely, the 3-variable Logistic LASSO showcased a structural robustness. Under 15% noise corruption, the most parsimonious model maintained a solid AUC of 0.794 (dropping only from 0.836).

These findings support the stability of the parsimonious model under simulated real-world measurement variability.

## 4. Discussion

In this study, we developed and internally tested a minimalist, clinically interpretable model for early risk stratification of patients with a higher probability of *Aspergillus* detection within a clinical context compatible with IPA in non-neutropenic ICU patients. The main finding is that a parsimonious three-variable model, including pulmonary galactomannan, non-specific pulmonary infiltrates, and age ≥ 65 years, achieved good discriminatory performance while maintaining clinical transparency. This triad reflects three complementary dimensions of IPA suspicion: biological evidence of fungal activity, radiological evidence of lung involvement, and host vulnerability.

The clinical relevance of this finding lies in the diagnostic uncertainty that characterizes IPA in critically ill, non-neutropenic patients. In this population, classical host factors and typical radiological signs are often absent, while invasive diagnostic procedures may be unsafe or impractical. Consequently, clinicians are frequently required to make treatment decisions based on incomplete and indirect evidence. A model relying on routinely available variables may therefore provide practical support for bedside decision-making. In particular, this model may help clinicians identify patients who warrant closer diagnostic evaluation, including repeat mycological testing, additional imaging, bronchoscopy when feasible, and infectious diseases consultation. However, the model should not be used to justify initiation of empirical antifungal therapy. Treatment decisions should continue to integrate disease severity, the overall clinical trajectory, competing diagnoses, microbiological findings, potential treatment-related toxicity, local epidemiology, and clinical judgement.

A key strength of the final model is its clinical coherence. Galactomannan represents a biologically plausible marker of fungal burden or active fungal growth. Non-specific pulmonary infiltrates provide the morphological context in which a positive mycological signal becomes clinically meaningful. Age, finally, captures a dimension of host vulnerability that may be particularly relevant in ICU patients, where immune dysfunction is often multifactorial and not fully represented by traditional definitions of immunosuppression. The combination of these three variables therefore provides a simple but clinically meaningful risk profile: a fungal biomarker, a radiological abnormality, and a host-related susceptibility factor.

An important observation of this study is that traditional systemic immunosuppression did not effectively stratify IPA risk when considered alone. This finding is clinically relevant because it supports the concept that IPA in non-neutropenic ICU patients should not be interpreted through the same framework used for hematological or profoundly immunocompromised populations [16,17]. In critically ill patients, susceptibility to IPA may result from a more complex interaction between host factors like advanced age, severe illness, lung injury, viral infections, corticosteroid exposure, organ dysfunction, and ICU-related immune dysregulation. Therefore, the absence of classical immunosuppressive conditions should not be considered reassuring when other biological and radiological signals are present.

The role of PCR deserves specific consideration. In univariate analysis, PCR was significantly associated with the outcome and showed clear diagnostic relevance. However, in the multivariable penalized framework, PCR provided limited incremental discriminatory value once Galactomannan, pulmonary infiltrates, and age were included. This does not imply that PCR is clinically irrelevant. Rather, it suggests that, in this cohort, PCR may have captured a partially overlapping or less stable signal, likely reflecting partial redundancy with other biological and radiological variables rather than an independent predictive contribution. A possible explanation is that PCR detects *Aspergillus* DNA but does not always distinguish between colonization, transient airway contamination, residual non-viable fungal material, and true invasive disease. In contrast, Galactomannan, particularly when interpreted together with compatible radiological abnormalities, may better reflect biologically active disease. Therefore, PCR should probably be interpreted as part of a broader diagnostic context rather than as an isolated decision-making variable. Moreover, both *Aspergillus* PCR and galactomannan are imperfect diagnostic tests, with recognized false-positive and false-negative results depending on specimen type, fungal burden, prior antifungal exposure, and the clinical setting. The proposed model should therefore not be interpreted as validating either biomarker individually. Instead, it integrates microbiological findings with radiological and host-related variables, thereby reducing reliance on any single diagnostic test.

The clustering analysis further supports the clinical applicability of the model. The high-risk cluster was characterized by the convergence of the three selected pillars: older age, pulmonary infiltrates, and galactomannan positivity. This group had a much higher observed rate of culture positivity, suggesting that the model may help identify patients who should undergo closer diagnostic evaluation or earlier consideration of antifungal treatment. In contrast, the low-risk group had a very low rate of culture positivity, suggesting that the model may also help avoid unnecessary antifungal treatment in patients with low predicted risk. This is clinically important because overtreatment is not harmless: antifungal therapy may be associated with toxicity, drug–drug interactions, costs, and selection pressure.

From a methodological perspective, the study also highlights the value of parsimony in small clinical datasets. More complex models and engineered interaction terms initially appeared promising, but their contribution was limited by collinearity and coefficient instability. The final LASSO model preserved or improved discrimination while reducing the features to three clinically interpretable variables. This supports the idea that, in low-prevalence conditions such as IPA, additional variables do not necessarily improve generalizability and may instead introduce noise, especially when the number of positive events is limited. Recent studies have applied predictive modeling to IPA in non-neutropenic patients using BALF-targeted next-generation sequencing [18] or complex machine-learning architectures [19]. Our study complements these approaches by showing that a clinically interpretable three-variable model, based only on galactomannan, pulmonary infiltrates, and age, can provide good discrimination while remaining applicable in settings where advanced molecular diagnostics are unavailable.

Nevertheless, several limitations must be acknowledged. First, this was a retrospective, monocentric study conducted in a specialized infectious disease ICU. The local epidemiology, patient case-mix, diagnostic practices, radiological interpretation, and laboratory procedures may differ from those of general ICUs or other hospital networks. As a result, external validity remains uncertain, and the model should not yet be considered ready for direct clinical implementation without prospective multicenter validation. In addition, owing to the retrospective nature of the study, several potentially relevant clinical variables, including lifestyle-related factors (such as smoking history, alcohol consumption, physical activity), detailed comorbidity profiles, and other laboratory parameters, were not consistently available in the electronic medical records and therefore could not be systematically incorporated into the analysis. Future prospective studies should evaluate the incremental contribution of these variables to the proposed model.

Second, the number of culture-positive cases was limited. Although the total cohort included 298 patients, only 25 were culture-positive, reflecting the low prevalence and diagnostic complexity of IPA in this population. This small number of events restricts the statistical power of the analysis, increases uncertainty around coefficient estimates, and limits the ability to evaluate more complex interactions. The use of nested CV and penalized regression reduces the risk of overfitting, but it cannot fully compensate for the limited number of outcome events, especially given the number of initial features considered together with the additional engineered ones, which outnumber the culture-positive patients. Another consequence of the limited number of culture-positive subjects is the use of the whole dataset for preprocessing steps, which can make our estimates optimistic and possibly introduce data leakage. Moreover, the outcome definition was based on culture positivity. While culture provides direct microbiological evidence of *Aspergillus* recovery, it does not necessarily prove tissue invasion. In ICU patients, *Aspergillus* isolation may reflect a spectrum ranging from colonization to invasive disease. Conversely, culture may lack sensitivity and may miss true IPA cases. Therefore, the selected reference standard may have introduced misclassification in both directions. Accordingly, the model should be interpreted as identifying a probability of microbiological positivity within a clinical context compatible with IPA, rather than definitive invasive disease. Future studies should consider adjudicated diagnostic endpoints based on internationally accepted definitions, integrating clinical, radiological, microbiological, and possibly histopathological criteria when available.

Third, radiological variables may be subject to interobserver variability. Non-specific pulmonary infiltrates are clinically meaningful but not pathognomonic, and their interpretation can vary according to imaging modality, timing, radiologist expertise, and the presence of concomitant lung diseases. This is particularly relevant in ICU patients, where atelectasis, bacterial pneumonia, ARDS (Acute Respiratory Distress Syndrome), pulmonary edema, and viral pneumonia may produce overlapping imaging patterns. Future validation should assess whether the model remains stable when radiological variables are reviewed across multiple centers and observers.

Fourth, the model was internally simulated but not externally validated. External validation in independent cohorts is necessary to determine calibration, discrimination, and clinical usefulness across different ICU populations. The introduction of calibration is particularly relevant as probability calibration may increase the clinical interpretability of the proposed model. However, the absence of external validation using datasets with a larger number of events also limits the assessment of the model’s robustness and its susceptibility to overfitting. For this reason, further testing should be conducted with high-population datasets to prove repeatability of the proposed methodology.

Finally, this model should be interpreted as a decision-support tool rather than a diagnostic rule. Its purpose is not to establish or exclude IPA independently, but to support clinical reasoning by identifying patterns of higher or lower probability. Because class weighting rescales the model’s intercept to handle class imbalance, predicted probability outputs should be interpreted as relative risk indices for bedside stratification rather than absolute diagnostic probabilities. The model may help clinicians prioritize further testing, repeat imaging, bronchoscopy when feasible, or early antifungal therapy. However, treatment decisions should continue to integrate the full clinical picture, including severity of illness, trajectory of respiratory failure, microbiological data, antifungal exposure, competing diagnoses, and local epidemiology.

## 5. Conclusions

In conclusion, this study suggests that early bedside risk stratification in non-neutropenic ICU patients undergoing diagnostic evaluation for suspected IPA may be supported by a simple and interpretable model based on pulmonary galactomannan positivity, lung involvement with the presence of non-specific pulmonary infiltrates, and advanced age. The findings support the potential clinical value of integrating biological, radiological, and host-related information rather than relying on isolated markers. However, the proposed model should be regarded as exploratory and proof of concept, as it was developed and internally validated within a single-center retrospective cohort using fungal culture positivity as the microbiological outcome. In this cohort of patients, the model demonstrated promising internal performance and practical interpretability, but its performance and clinical utility remain to be established through prospective, multicenter external validation using independently adjudicated IPA endpoints before any clinical implementation. Future studies should assess model calibration and clinical utility and determine whether model-guided risk stratification can improve diagnostic pathways, antifungal stewardship, appropriateness of therapy, and patient outcomes.

## Figures and Tables

**Table 4:** Evolution of LASSO model performance and coefficient stability during the iterative pruning process. The transition to the 3-variable model maximizes AUC while achieving minimal residual collinearity (VIF ≈ 1.0).

Model 1	Features	AUC ± SD	Sensitivity	Specificity	^a^ LR+	^b^ LR−	^c^ PPV (%)	^d^ NPV (%)	Max VIF	Dropped/Weak Coefficients
Phase 1 (Baseline)	8	0.822 ± 0.071	0.600 ± 0.141	0.766 ± 0.088	2.56	0.52	19.0	95.3	6.74 (Critical)	both FE_* and halo_sign
Phase 2 (Intermediate)	5	0.825 ± 0.088	0.720 ± 0.110	0.729 ± 0.063	2.66	0.38	19.6	96.6	3.10 (Warning)	PCR and air_crescent_sign
Phase 3 (Final)	3	0.836 ± 0.075	0.720 ± 0.110	0.740 ± 0.059	2.77	0.38	20.2	96.8	1.07 (Optimal)	None (All robust)

^a^ LR+, Positive Likelihood Ratio. ^b^ LR−, Negative Likelihood Ration. ^c^ PPV, Positive Predictive Value. ^d^ NPV, Negative Predictive Value. * FE, Feature Engineering.

**Table 5:** Ablation and substitution sensitivity analysis. The minimalist consistently outperforms all configurations involving PCR or reduced features.

Configuration	Features	AUC	Sensitivity	Specificity
Minimalist	Gal, Inf, Age	0.836 ± 0.075	0.720 ± 0.11	0.740 ± 0.059
Without Gal	PCR, Inf, Age	0.778 ± 0.082	0.720 ± 0.11	0.740 ± 0.097
Without Inf	Gal, PCR, Age	0.809 ± 0.037	0.600 ± 0.245	0.703 ± 0.069
Without Age	Gal, Inf	0.796 ± 0.099	0.840 ± 0.089	0.667 ± 0.065
Gal and PCR	Gal, PCR	0.787 ± 0.082	0.720 ± 0.303	0.644 ± 0.103

**Table 6:** Clinical Risk Stratification under the 3-Variable.

Cluster	N	True Positives	Clinical Profile	Prevalence (Real)
Low Risk	98	0	Gal (0%), Inf (0%), Age (0%)	0.0%
Medium Risk	123	8	Age (72%), Inf (19%), Gal (10%)	6.5%
High Risk	77	17	Age (88%), Inf (81%), Gal (56%)	22.1%

**Table 7:** Comparative performance of experimental models highlighting the transition from the 8-variable full set to the 3-variable across 5 Nested CV folds.

Model Architecture	ROC-AUC ± SD		Sensitivity ± SD		Specificity ± SD	
	8 Vars	3 Vars	8 Vars	3 Vars	8 Vars	3 Vars
PCA + SVM *	0.565 ± 0.244	0.660 ± 0.136	0.600 ± 0.141	0.440 ± 0.219	0.725 ± 0.052	0.787 ± 0.039
XGBoost	0.723 ± 0.135	0.791 ± 0.072	0.520 ± 0.179	0.680 ± 0.110	0.806 ± 0.087	0.762 ± 0.068
Random Forest	0.836 ± 0.074	0.773 ± 0.072	0.560 ± 0.329	0.640 ± 0.089	0.806 ± 0.096	0.769 ± 0.066
Hybrid RF Ensemble	0.834 ± 0.076	0.812 ± 0.096	0.560 ± 0.329	0.680 ± 0.110	0.809 ± 0.099	0.765 ± 0.072
Logistic LASSO	0.822 ± 0.071	0.836 ± 0.075	0.600 ± 0.141	0.720 ± 0.110	0.766 ± 0.088	0.740 ± 0.059

* Reported values refer to directions detected by PCA.

## Data Availability

The data supporting the findings of this study are available upon rea-sonable request. Due to the sensitive nature of the clinical information derived from hospitalized patients, the dataset and code cannot be made publicly accessible. The corresponding authors may provide anonymized data upon justified and reasonable request. No protocol was prepared for this study.

## Supplementary Materials

The following supporting information can be downloaded at https://www.mdpi.com/article/10.3390/jof12090658/s1, Supplementary Tables S1: Binarization thresholds employed in the processing of the dataset. The original dataset was an Excel produced by the ICU ward according to full patient history during their stay at the time of their dismissal; Table S2: Schematic of the intercept values depending on intercept scale; Table S3: Schematic of the coefficients over different intercept scales; Table S4: Probabilities for each combination of main binary variables; Table S5: Univariate associations and Bonferroni-adjusted significance threshold for candidate predictors; Table S6: Sensitivity, specificity, PPV and NPV obtained over different thresholds; Table S7: Confidence intervals and out-of-fold AUC for the tested variations of the model; Table S8: Paired comparison of the minimalist model with other tested configurations of features; Table S9: TRIPODAI Checklist; Figure S1: PCA biplot representation of the full 25-variable configuration. The plot illustrates the global covariance structure of the dataset; the spatial overlap between Col positive and Col negative classes highlights the inherent complexity in establishing an unsupervised decision boundary. Refs. [20,21] are cited in Supplemental Materials.

## Abbreviations

The following abbreviations are used in this manuscript:

IPA	Invasive Pulmonary Aspergillosis
ICU	Intensive Care Unit
AUC	Area Under the Curve
Gal	Galactomannan
Col	Culture
Inf	non-specific Infiltrates
Immuno	Immunosuppressive status
FE	Feature Engineering
OR	Odds Ratio
VIF	Variance Inflation Factor
HSCT	Hematopoietic Stem Cell Transplantation
NCV	Nested Cross-Validation
