# Supplementary material for: Early Bedside Risk Stratification in ICU Patients with Suspected Invasive Pulmonary Aspergillosis: A Minimalist Clinical Decision Support Model
Source: J Fungi (Basel). 2026 Sep 2;12(9):658. doi: 10.3390/jof12090658 (PMC13608610; doi:10.3390/jof12090658)
Supplement: Supplementary file 1 [file jof-12-00658-s001.zip › jof-4446568-supplementary.pdf]

Supplementary Material

S1. Binarization Thresholds

Supplementary Table S1 summarizes the full array of binarization thresholds used in our work to interpret clinical results. As evidenced by the binarization thresholds, the majority of included variables were already pre-binarized in the database as they depend on the simple presence of a specific pathology or radiological sign. For the rest of non-binarized variables, the majority of values depended on the presence of any positivity in the database (e.g., history of organ transplant, hematological or oncological malignancies, meningitis and encephalitis), whereas the laboratory markers were evaluated based on the results of laboratory analyses, leading to a direct binarization. The only true threshold employed was for age, which as mentioned in the main submission was selected based on clinical interpretability of old age. Binarization of the ground truth (i.e., culture positivity) was performed, similarly to laboratory markers, depending on laboratory results, with positivity being determined if positivity to *Aspergillus* was detected in at least one analysis. All variables were binarized with values 0/1.

Supplementary Table S1. Binarization thresholds employed in the processing of the dataset. The original dataset was an Excel produced by the ICU ward according to full patient history during their stay at the time of their dismissal.

Variable	Database value	Binarization method
	Host factors	
Sex	1 for male, 2 for female	0 for male, 1 for female
Age	Numerical value	1 for age ≥ 65, 0 otherwise
Use of corticosteroids	0 if not used, 1 if used	Already binarized
Treatment with cytotoxic agents	0 if not used, 1 if used	Already binarized
History of organ transplant	0 if no transplant, transplanted organ name if transplant occurred	0 if no transplant, 1 if any transplant occurred
HIV	0 if not present, 1 if present	Already binarized
Hematological/oncological malignancies	0 if not present, malignancy name if present	0 if not present, 1 if any malignancy present
Immunosuppression	0 if not present, 1 if present	Already binarized
Arterial hypertension	If present, marked as IA among comorbidities	0 if not present, 1 if present
Diabetes mellitus	If present, marked as DM among comorbidities	0 if not present, 1 if present
COPD	If present, marked as BPCO among comorbidities	0 if not present, 1 if present
COVID-19 infection	0 if not positive, 1 if positive	Already binarized
Meningitis and encephalitis	Pathology name in 'OTHER' field if present	0 if not present, 1 if any pathology present
Refractory respiratory failure despite mechanical ventilation	0 if not occurred, 1 if occurred	Already binarized
	Radiological signs	
Air crescent sign	0 if not present, 1 if present	Already binarized
Cavity	0 if not present, 1 if present	Already binarized
Circumscribed lesion with or without halo sign	0 if not present, 1 if present	Already binarized
Alveolar or reticular opacities	0 if not present, 1 if present	Already binarized

Non-specific pulmonary infiltrates	0 if not present, 1 if present	Already binarized
Pleural effusion	0 if not present, 1 if present	Already binarized
Wedge-shaped opacities	0 if not present, 1 if present	Already binarized
Tree-in-bud pattern	0 if not present, 1 if present	Already binarized
	Laboratory markers	
PCR	History over max 5 samples, specifying sample type and date	1 if *Aspergillus* DNA is found, 0 otherwise
Galactomannan (gal)	History over max 4 samples, specifying sample type and date	1 if gal index ≥ 0.5, 0 otherwise
	Ground truth	
Culture	History over max 7 samples, specifying date and quantity of colonies (0 if none)	1 if *Aspergillus* colonies found at least once, 0 otherwise

S2. Intercept

The intercept exists on two scales, shown in Supplementary Table S2. The two values are not interchangeable. The first one only works with standardized variables, while the second one only with 0/1 variables. The binarized scale intercept is the one used in our final equation. For completeness, the intercepts of the five outer-fold models on the original scale are −1.8771, −1.2495, −1.0271, −1.6674 and −1.8597. The spread is in line with the spread of the coefficients already reported in the paper.

Supplementary Table S2. Schematic of the intercept values depending on intercept scale.

**Intercept scale**	**Value**
Standardized scale	-0.3563
Original 0/1 scale	-1.2456

S3. Coefficients

Similarly to the intercept, we report the coefficients for both scales (standardized and binarized) in Supplementary Table S3 with the corresponding odd ratios. Two clarifications are needed. The first is that the standardized coefficients are used for the LASSO model fitting and are therefore reported for reproducibility of the fitting procedure. Signs and ordering are identical on both scales, so nothing in the interpretation changes. Such coefficients should never be combined with binarized inputs, which are instead used in the final equation to score new patients. The second is that the ± values represent the spread across the five outer folds, not standard errors of the estimate. On the original scale, the fold-to-fold ranges are gal 1.24–1.97, inf 0.90–1.91, age 0.62–0.79. No variable changes sign in any fold, which supports the stability of the triad.

Supplementary Table S3. Schematic of the coefficients over different intercept scales.

**Variable**	**Standardized (final model)**	**Original 0/1 scale**	**Odds ratio**
(intercept)	-0.3563	-1.2456	--
Gal	+0.608 ± 0.110	+1.4094	4.09
Non-specific infiltrates	+0.615 ± 0.160	+1.2250	3.40
Age (≥ 65)	+0.350 ± 0.033	+0.5344	1.71

S4. Probabilities and Final Equation

To compute the probabilities, we apply our model's logistic function to the linear predictor. The final model is a logistic regression with L1 (LASSO) penalty on the three selected variables. The model was trained on standardized variables, so the coefficients reported in Section 4.2.3 are on the standardized scale, not on the original 0/1 scale. On the original 0/1 scale, the final equation is:

$$z = -1.2456 + 1.4094 * gal + 1.2250 * inf + 0.5344 * age$$

$$P = 1 / (1 + exp(-z))$$

where gal = 1 if pulmonary galactomannan is positive, inf = 1 if non-specific pulmonary infiltrates are present, and age = 1 if the patient is 65 or older. The probabilities computed manually with this formula match the ones produced by the trained model exactly (difference in the order of 10^{-16}).

Since the three variables are binary, the whole model fits in an eight-row table, shown in Supplementary Table S4. The model uses balanced class weights as described in Section 4. The reason for the choice is to protect sensitivity in the minority class, as it rebalances the two classes to 1:1 during fitting, so the output is a risk score calibrated on balanced prevalence, not an absolute probability of disease. That is why the values in the table sit systematically above the observed prevalence in the same cells. Furthermore, the equation is from the model refitted on the full cohort of 298 patients. This is the standard way to obtain a model for distribution: the nested cross-validation estimates performance without optimism, while the final model is refitted on all available data using the same internal procedure for selecting C. The reported AUC of 0.836 remains the nested-CV estimate and is not in-sample.

Supplementary Table S4. Probabilities for each combination of main binary variables.

gal	inf	age	z	P (final model)	N	Observed positives
0	0	0	-1.2456	0.224	98	0
0	0	1	-0.7112	0.329	88	4
0	1	0	-0.0206	0.495	23	2
1	0	0	+0.1637	0.541	12	2
0	1	1	+0.5138	0.626	34	4
1	0	1	+0.6982	0.668	15	1
1	1	0	+1.3888	0.800	9	2
1	1	1	+1.9232	0.873	19	10

To understand absolute risks, we also provide the unweighted version of the same model, which keeps identical signs and identical ranking of patients but is centered on the true 8.4% prevalence:

$$logit(p) = -3.7769 + 1.5494 * gal + 1.3725 * inf + 0.5973 * age$$

giving 0.022, 0.040, 0.083, 0.097, 0.141, 0.164, 0.298 and 0.436 for the eight profiles. These agree well with the observed prevalences in Table 10.

S5. Bonferroni Correction

The univariate screening (odds ratios, p-values, tiering) was run once on the full cohort of 298 patients, before cross-validation. The threshold of $p < 0.00179$ reported in Section 4 is 0.05/28, where 28 is the number of variables tested after feature engineering. The Bonferroni threshold was used as a descriptive reference when presenting the univariate results. The tiering rules that actually drove variable selection used uncorrected thresholds (OR > 3 and $p < 0.01$ for Tier 1, OR > 2 and $p < 0.05$ for Tier 2). The corrected values are shown in Supplementary Table S5, indicating whether each feature's p-value is under the defined threshold.

Supplementary Table S5. Univariate associations and Bonferroni-adjusted significance threshold for candidate predictors.

Feature	OR	*p*-value	p < 0.00179
FE_Inf_x_Gal	14.8	< 0.001	passes
FE_Gal_x_PCR	10.7	< 0.001	passes
Gal	8.7	< 0.001	passes
Inf	7.9	< 0.001	passes
PCR	7.1	< 0.001	passes
Air crescent sign	5.4	0.0086	does not pass
Age (≥ 65 years)	3.1	0.0198	does not pass
Halo sign	2.8	0.0220	does not pass

The two central variables of the final model, galactomannan and non-specific infiltrates, pass the correction comfortably. Age does not pass at the univariate due to its multivariate contribution, which is supported by the ablation study in Section 4.2.4: removing age lowers AUC from 0.836 to 0.796 and specificity from 0.740 to 0.667, and the variable keeps a positive, stable coefficient in all five folds, with the smallest spread of the three (SD 0.033). This is the same reasoning applied for PCR and galactomannan, which were kept as primary biomarkers regardless of their tier.

S6. Risk Categories Thresholds

The Low / Medium / High stratification in Section 4.2.5 groups patients by the risk score produced by the model, rather than by thresholds fixed in advance. Patients are partitioned into three groups on the basis of the model score, and each group is then characterized afterwards by its prevailing clinical profile and by the IPA prevalence actually observed in it. The equivalent thresholds on the model score are $p < 0.206$ for low risk, $0.206 \leq p < 0.555$ for medium risk and $p \geq 0.555$ for high risk. Because the three variables are binary, these thresholds are reduced to a simple bedside rule. We first count how many out of galactomannan, non-specific Infiltrates and age are positive and then assign a risk level based on the number of positives (0 → low risk, 1 → medium risk, 2 or 3 → high risk).

The sensitivity of 0.72 and specificity of 0.74 reported in the abstract correspond to a decision threshold of 0.5 on the model score. Performance at two other clinically relevant thresholds, computed on out-of-fold predictions as shown in Supplementary Table S6. The NPV above 0.96 at every threshold is clinically the most relevant result of the stratification, as the low-risk group contains no IPA case in this cohort.

Supplementary Table S6. Sensitivity, specificity, PPV and NPV obtained over different thresholds.

Rule	Threshold	Sensitivity	Specificity	PPV	NPV
Threshold used in the paper	0.500	0.720	0.740	0.202	0.967
Maximum Youden index	0.412	0.840	0.667	0.188	0.978
Rule-out oriented (Sens ≥ 0.90)	0.265	0.920	0.465	0.136	0.984

S7. Results

S7.1 Iterative model refinement and feature selection

S7.1.1 Phase 1: The 8-Variable Baseline Model

The initial LASSO execution was performed using the 8 top-tier aforementioned variables identified during the Exploratory Data analysis (EDA) (Table 4, main text).

The aggregated performance across the 5 outer folds of the Nested Cross-Validation (NCV) yielded an AUC of 0.822 ± 0.071, with a Sensitivity of 0.600 ± 0.141 and a Specificity of 0.766 ± 0.088. While the ROC-AUC appeared acceptable, the internal mechanics of the model revealed significant structural instability.

Engineered interactions FE_Gal_x_PCR and FE_inf_x_Gal registered critical Variance Inflation Factor (VIF) scores (6.74 and 4.76, respectively). Pearson correlation confirmed a massive overlap between the interaction FE_Gal_x_PCR and the base gal variable (r = 0.804). Remarkably, the LASSO penalty assigned conflicting weights: while Gal was a strong positive predictor (+0.623 ± 0.104), the interaction FE_Gal_x_PCR received a negative coefficient of −0.091 ± 0.168 in an attempt to mathematically offset the base variable's weight.
This phase demonstrated that without human pruning, the model uses interactions as mathematical counterweights rather than biological signals.

S7.1.2 Phase 2: The 5-Variable Intermediate Model

In the second iteration, we manually pruned the artificial interactions and the circumscribed lesion with or without halo sign (halo_sign) which showed inconsistent behavior and negative coefficients (−0.213 ± 0.124) despite its known clinical specificity. The model was retrained on a 5-variable subset: Gal, Inf, Age, PCR, and air_crescent_sign. The NCV evaluation showed an improvement in capture: sensitivity jumped from 0.600 to 0.720 ± 0.110, while maintaining a ROC-AUC of 0.825 ± 0.088. However, the coefficient stability analysis highlighted a persistent layer of "noise". The PCR variable presented a coefficient of +0.094, but with a standard deviation (±0.184) overcoming its own magnitude. Similarly, the air_crescent_sign received a weight of +0.031±0.034. VIF analysis confirmed that PCR and Inf were still locked in collinearity (r = 0.735), suggesting that the LASSO was retaining PCR just for local data fitting rather than universal pattern recognition.

S7.2 Feature substitution additional results

Two intervals per configuration have been additionally calculated. The first is the 95% CI of the mean across the five outer folds (t distribution, 4 degrees of freedom), which measures stability across folds. The second is a 95% bootstrap interval (2,000 stratified resamples) on the AUC computed from the pooled out-of-fold predictions, which is the interval that belongs next to a published AUC, shown in Supplementary Table S7. The minimalist configuration remains the best one, with the highest out-of-fold AUC (0.818) of every configuration tested. It must be noted that the intervals of different configurations overlap. On a single evaluation, these numbers show a consistent advantage for the triad, but not a clean statistical separation from each alternative taken individually.

Supplementary Table S7. Confidence intervals and out-of-fold AUC for the tested variations of the model.

Configuration	Features	95% CI (folds)	Out-of-fold AUC [95% boot.]
Minimalist	Gal, Inf, Age	[0.743, 0.929]	0.818 [0.730, 0.897]
Without Gal	PCR, Inf, Age	[0.676, 0.880]	0.753 [0.647, 0.850]
Without Inf	Gal, PCR, Age	[0.763, 0.854]	0.796 [0.705, 0.877]
Without Age	Gal, Inf	[0.674, 0.919]	0.773 [0.663, 0.869]
Gal and PCR	Gal, PCR	[0.685, 0.889]	0.775 [0.673, 0.865]
Phase 2	5 variables	[0.717, 0.934]	0.796 [0.697, 0.886]
Phase 1	8 variables	[0.734, 0.910]	0.811 [0.730, 0.885]

For this reason, we verified the results of the minimalist model with a paired comparison with the other configurations. We repeated the entire nested cross-validation on 20 different fold partitions and compared configurations on the same partitions. The results are shown in Supplementary Table S8. The minimalist configuration wins in 19 or 20 partitions out of 20 against every alternative. This shows its selection is not an artifact of the particular fold partition used in the paper. The 20 repetitions run on the same cohort, establishing robustness with respect to data partitioning rather than inferential superiority in the general population, with the latter requiring the prospective multicenter validation suggested in the conclusions. For transparency,

across the 20 partitions the mean AUC of the minimalist configuration is 0.820 ± 0.015, so the 0.836 reported in the paper sits in the upper part of that distribution.

Supplementary Table S8. Paired comparison of the minimalist model with other tested configurations of features.

Alternative configuration	Features	Times it beats the minimalist	Mean AUC difference
Without Gal	PCR, Inf, Age	0/20	-0.065
Without Inf	Gal, PCR, Age	0/20	-0.018
Without Age	Gal, Inf	1/20	-0.022
Gal and PCR	Gal, PCR	0/20	-0.025
Phase 2	5 variables	0/20	-0.022
Phase 1	8 variables	0/20	-0.042

S7.3 PCA-based visualization and model comparison

Exploratory benchmarking analyses were performed across the 8-variable, 5-variable, and 3-variable feature configurations to assess the stability of model performance under progressive feature reduction. The evaluated architectures included Random Forest, XGBoost, PCA followed by SVM, and a LASSO-guided Random Forest ensemble. The application of PCA combined with the SVM model did not return the highest predictive performance; however, the projection offers significant clinical interpretability.

As shown in Figure S1, the PCA visualizes the entire initial feature set, revealing compelling dynamics between key predictors. Several significant parameters do not emerge as drivers of variance within the first two principal components. In particular, Gal and PCR vectors projected in a similar direction, supporting biological coherence between these mycological markers. Nevertheless, PCR showed a greater magnitude than Gal in the PCA space. This finding should be interpreted cautiously, as PCA only focuses on 'simple' variance which is less informative unlike other supervised models which incorporate class weighting to account for class imbalance, giving greater influence on the underrepresented positive class during model training. Therefore, the PCA projection was considered mainly exploratory and hypothesis-generating rather than confirmatory of predictor importance.

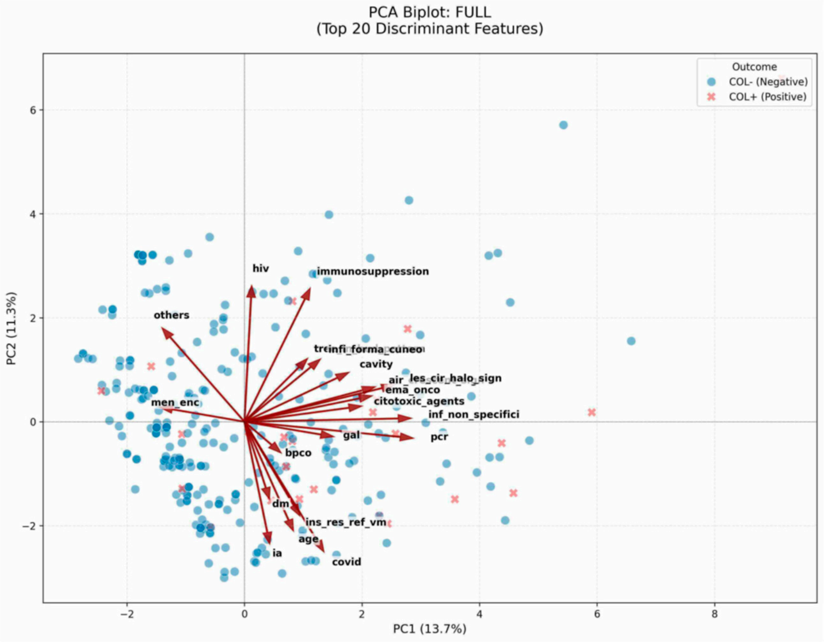

Figure S1. PCA biplot representation of the full 25-variable configuration. The plot illustrates the global covariance structure of the dataset; the spatial overlap between Col positive and Col negative classes highlights the inherent complexity in establishing an unsupervised decision boundary.

S7.3 TRIPODAI Checklist

Supplementary Table S9. TRIPODAI Checklist.

TRIPOD+AI

Version: 11-January-2024

Section/Topic	Item	Development / evaluation[1]	Checklist item	Reported on page
**TITLE**				
Title	1	D;E	Identify the study as developing or evaluating the performance of a multivariable prediction model, the target population, and the outcome to be predicted	Title/Abstract, pp.1
**ABSTRACT**				
Abstract	2	D;E	See TRIPOD+AI for Abstracts checklist	Abstract, pp.1
**INTRODUCTION**				
Background	3a	D;E	Explain the healthcare context (including whether diagnostic or prognostic) and rationale for developing or evaluating the prediction model, including references to existing models	Section 1, pp.2
	3b	D;E	Describe the target population and the intended purpose of the prediction model in the context of the care pathway, including its intended users (e.g., healthcare professionals, patients, public)	Section 1, pp.2-3
	3c	D;E	Describe any known health inequalities between sociodemographic groups	Not reported
Objectives	4	D;E	Specify the study objectives, including whether the study describes the development or validation of a prediction model (or both)	Section 1, pp.3
**METHODS**				
Data	5a	D;E	Describe the sources of data separately for the development and evaluation datasets (e.g., randomised trial, cohort, routine care or registry data), the rationale for using these data, and representativeness of the data	Section 2.1-2.2, pp.4
	5b	D;E	Specify the dates of the collected participant data, including start and end of participant accrual; and, if applicable, end of follow-up	Section 2.2, pp.4
Participants	6a	D;E	Specify key elements of the study setting (e.g., primary care, secondary care, general population) including the number and location of centres	Section 2.1-2.2, pp.4
	6b	D;E	Describe the eligibility criteria for study participants	Section 2.2, pp.4
	6c	D;E	Give details of any treatments received, and how they were handled during model development or evaluation, if relevant	Section 2.5, pp.5
Data preparation	7	D;E	Describe any data pre-processing and quality checking, including whether this was similar across relevant sociodemographic groups	Section 2.4-2.6, pp.4-6, Supp Section 1-6, pp.1-5
Outcome	8a	D;E	Clearly define the outcome that is being predicted and the time horizon, including how and when assessed, the rationale for choosing this outcome, and whether the method of outcome assessment is consistent across sociodemographic groups	Section 2.2, 2.5, pp. 4, 5
	8b	D;E	If outcome assessment requires subjective interpretation, describe the qualifications and demographic characteristics of the outcome assessors	Section 2.2-2.3, pp.4
	8c	D;E	Report any actions to blind assessment of the outcome to be predicted	Not reported
Predictors	9a	D	Describe the choice of initial predictors (e.g., literature, previous models, all available predictors) and any pre-selection of predictors before model building	Section 2.4-2.6, pp.4-6
	9b	D;E	Clearly define all predictors, including how and when they were measured (and any actions to blind assessment of predictors for the outcome and other predictors)	Section 2.4-2.6, pp.4-6, Supp Section 1-6, pp.1-5
	9c	D;E	If predictor measurement requires subjective interpretation, describe the qualifications and demographic characteristics of the predictor assessors	Section 2.2, 4, pp.4, 14
Sample size	10	D;E	Explain how the study size was arrived at (separately for development and evaluation), and justify that the study size was sufficient to answer the research question. Include details of any sample size calculation	Section 1, 2.7, pp.2, 6
Missing data	11	D;E	Describe how missing data were handled. Provide reasons for omitting any data	Section 2.4, pp.4-5
Analytical methods	12a	D	Describe how the data were used (e.g., for development and evaluation of model performance) in the analysis, including whether the data were partitioned, considering any sample size requirements	Section 2.7-2.8, pp.6-7
	12b	D	Depending on the type of model, describe how predictors were handled in the analyses (functional form, rescaling, transformation, or any standardisation).	Section 2.4, 2.8, pp.4-5, 6-7, Supp Section 1-6, pp.1-5
	12c	D	Specify the type of model, rationale[2], all model-building steps, including any hyperparameter tuning, and method for internal validation	Section 2.8, 3.1, pp. 6-9
	12d	D;E	Describe if and how any heterogeneity in estimates of model parameter values and model performance was handled and quantified across clusters (e.g., hospitals, countries). See TRIPOD-Cluster for additional considerations[3]	Not applicable (single-center study)
	12e	D;E	Specify all measures and plots used (and their rationale) to evaluate model performance (e.g., discrimination, calibration, clinical utility) and, if relevant, to compare multiple models	Section 2.8, p p.6-7
	12f	E	Describe any model updating (e.g., recalibration) arising from the model evaluation, either overall or for particular sociodemographic groups or settings	Not applicable (no update)
	12g	E	For model evaluation, describe how the model predictions were calculated (e.g., formula, code, object, application programming interface)	Supp Section 4, pp.3-4
Class imbalance	13	D;E	If class imbalance methods were used, state why and how this was done, and any subsequent methods to recalibrate the model or the model predictions	Section 2.8, 3.1, pp.6-9
Fairness	14	D;E	Describe any approaches that were used to address model fairness and their rationale	Not examined
Model output	15	D	Specify the output of the prediction model (e.g., probabilities, classification). Provide details and rationale for any classification and how the thresholds were identified	Section 3.3, 3.5, pp. 9-10, 11-12, Supp Section 4, 6, pp. 3-4, 4-5

[1] D=items relevant only to the development of a prediction model; E=items relating solely to the evaluation of a prediction model; D;E=items applicable to both the development and evaluation of a prediction model

[2] Separately for all model building approaches.

[3] TRIPOD-Cluster is a checklist of reporting recommendations for studies developing or validating models that explicitly account for clustering or explore heterogeneity in model performance (eg, at different hospitals or centres). Debray et al, BMJ 2023; 380: e071018 [DOI: 10.1136/bmj-2022-071018]

Training versus evaluation	16	D;E	Identify any differences between the development and evaluation data in healthcare setting, eligibility criteria, outcome, and predictors	Not applicable (internal NCV validation only)
Ethical approval	17	D;E	Name the institutional research board or ethics committee that approved the study and describe the participant-informed consent or the ethics committee waiver of informed consent	Section 2.3, pp.4 Appendix, pp.17
**OPEN SCIENCE**				
Funding	18a	D;E	Give the source of funding and the role of the funders for the present study	Appendix, pp. 17
Conflicts of interest	18b	D;E	Declare any conflicts of interest and financial disclosures for all authors	Appendix, pp.17
Protocol	18c	D;E	Indicate where the study protocol can be accessed or state that a protocol was not prepared	Appendix, pp.17
Registration	18d	D;E	Provide registration information for the study, including register name and registration number, or state that the study was not registered	Not reported
Data sharing	18e	D;E	Provide details of the availability of the study data	Appendix, pp.16
Code sharing	18f	D;E	Provide details of the availability of the analytical code[4]	Appendix, pp.16
**PATIENT & PUBLIC INVOLVEMENT**				
Patient & Public Involvement	19	D;E	Provide details of any patient and public involvement during the design, conduct, reporting, interpretation, or dissemination of the study or state no involvement.	Section 2.2, pp.4
**RESULTS**				
Participants	20a	D;E	Describe the flow of participants through the study, including the number of participants with and without the outcome and, if applicable, a summary of the follow-up time. A diagram may be helpful.	Section 1, 3.1, pp.2, 7-8
	20b	D;E	Report the characteristics overall and, where applicable, for each data source or setting, including the key dates, key predictors (including demographics), treatments received, sample size, number of outcome events, follow-up time, and amount of missing data. A table may be helpful. Report any differences across key demographic groups.	Section 3.1, pp.7-9
	20c	E	For model evaluation, show a comparison with the development data of the distribution of important predictors (demographics, predictors, and outcome).	Not applicable (internal NCV validation only)
Model development	21	D;E	Specify the number of participants and outcome events in each analysis (e.g., for model development, hyperparameter tuning, model evaluation)	Section 2.8, 3.1, pp.6-9
Model specification	22	D	Provide details of the full prediction model (e.g., formula, code, object, application programming interface) to allow predictions in new individuals and to enable third-party evaluation and implementation, including any restrictions to access or re-use (e.g., freely available, proprietary)[5]	Section 3.3, pp.9-10, Supp Section 4, pp.3-4
Model performance	23a	D;E	Report model performance estimates with confidence intervals, including for any key subgroups (e.g., sociodemographic). Consider plots to aid presentation.	Section 3.3, 3.7, pp.9-10, 12-13
	23b	D;E	If examined, report results of any heterogeneity in model performance across clusters. See TRIPOD Cluster for additional details[3].	Not examined
Model updating	24	E	Report the results from any model updating, including the updated model and subsequent performance	Not applicable (no update)
**DISCUSSION**				
Interpretation	25	D;E	Give an overall interpretation of the main results, including issues of fairness in the context of the objectives and previous studies	Section 4, pp.14-15
Limitations	26	D;E	Discuss any limitations of the study (such as a non-representative sample, sample size, overfitting, missing data) and their effects on any biases, statistical uncertainty, and generalizability	Section 4, pp.15-17
Usability of the model in the context of current care	27a	D	Describe how poor quality or unavailable input data (e.g., predictor values) should be assessed and handled when implementing the prediction model	Section 4, pp. 16
	27b	D	Specify whether users will be required to interact in the handling of the input data or use of the model, and what level of expertise is required of users	Section 1, 4, pp. 2, 14
	27c	D;E	Discuss any next steps for future research, with a specific view to applicability and generalizability of the model	Section 4, Conclusions, pp. 15-17

From: Collins GS, Moons KGM, Dhiman P, et al. *BMJ* 2024;385:e078378. doi:10.1136/bmj-2023-078378

[4] This relates to the analysis code, for example, any data cleaning, feature engineering, model building, evaluation.
[5] This relates to the code to implement the model to get estimates of risk for a new individual.
